# SHP-1 tyrosine phosphatase binding to c-Src kinase phosphor-dependent conformations: A comparative structural framework

**DOI:** 10.1371/journal.pone.0278448

**Published:** 2023-01-13

**Authors:** Mehreen Gul, Ahmad Navid, Muhammad Fakhar, Sajid Rashid

**Affiliations:** National Center for Bioinformatics, Quaid-i-Azam University, Islamabad, Pakistan; Hungarian Academy of Sciences, HUNGARY

## Abstract

SHP-1 is a cytosolic tyrosine phosphatase that is primarily expressed in hematopoietic cells. It acts as a negative regulator of numerous signaling pathways and controls multiple cellular functions involved in cancer pathogenesis. This study describes the binding preferences of SHP-1 (pY536) to c-Src^open^ (pY416) and c-Src^close^ (pY527) through *in silico* approaches. Molecular dynamics simulation analysis revealed more conformational changes in c-Src^close^ upon binding to SHP-1, as compared to its active/open conformation that is stabilized by the cooperative binding of the C-SH2 domain and C-terminal tail of SHP-1 to c-Src SH2 and KD. In contrast, c-Src^close^ and SHP-1 interaction is mediated by PTP domain-specific WPD-loop (WPDXGXP) and Q-loop (QTXXQYXF) binding to c-Src^close^ C-terminal tail residues. The dynamic correlation analysis demonstrated a positive correlation for SHP-1 PTP with KD, SH3, and the C-terminal tail of c-Src^close^. In the case of the c-Src^open^-SHP-1 complex, SH3 and SH2 domains of c-Src^open^ were correlated to C-SH2 and the C-terminal tail of SHP-1. Our findings reveal that SHP1-dependent c-Src activation through dephosphorylation relies on the conformational shift in the inhibitory C-terminal tail that may ease the recruitment of the N-SH2 domain to phosphotyrosine residue, resulting in the relieving of the PTP domain. Collectively, this study delineates the intermolecular interaction paradigm and underlying conformational readjustments in SHP-1 due to binding with the c-Src active and inactive state. This study will largely help in devising novel therapeutic strategies for targeting cancer development.

## Introduction

Protein tyrosine phosphorylation is a post-translational modification that plays an essential role in cell growth, proliferation, and differentiation [1, 2]. Tyrosine phosphorylation is a fundamental mechanism in the eukaryotic cellular signaling pathways [3]. The protein tyrosine phosphorylation level is rigorously controlled by two types of enzymes that exert opposite biological functions [4, 5] protein-tyrosine phosphatases (PTPs) and protein-tyrosine kinases (PTKs). PTPs counterbalance the PTK phosphorylation process through dephosphorylation of the phosphorylated tyrosine [5–7]. The structures and functions of PTKs have been widely studied compared to PTPs [8], which can be categorized into four groups, including receptor-like PTPs (RPTPs), non-receptor PTPs (non-TM PTPs), dual-specificity phosphatases (DSPs), and low molecular weight phosphatases (LMPs) [6]. SH2 domain-containing protein tyrosine phosphatases (SHP-1 and SHP-2) exhibit high sequence identity with each other by sharing two SH2 domains at the N-terminal region followed by a catalytic domain (PTP domain) and a C-terminal tail [4, 5].

SHP-1 contains 60% sequence identity with SHP-2, while at the structural level, C-SH2 domains of both SHPs are dissimilar and play a crucial role in phosphopeptide binding in triggering the enzymatic activity [9, 10]. SHP-1 is mainly expressed in hematopoietic cells and is commonly considered as a negative regulator of multiple signaling pathways [11–15]. SH2 domain binding initiates SHP-1 activation by recruiting it to the phosphorylated residues of the immune receptor tyrosine-based inhibitory motifs (ITIMs) of the innumerable activators for dephosphorylation of downstream targets [16]. SHP-1 activation is triggered by its tyrosine-phosphorylated tail [17] and acidic phospholipids [18]. Through *in vitro* studies, it has been reported that Src phosphorylates SHP-1 (Y536 and Y564 residues) by binding at the NH_2_-terminal of the SH2 domain in the lymphocytes and platelets cells [19–22]. The amalgamation of phosphonomethylenephenylalanine (Pmp) at the Y536-site induces 4-fold stimulation of SHP-1 tyrosine phosphatase action. Assimilation of another phosphonate analog difluorophosphonomethylenephenylalanine (F2Pmp) at Y536-site further enhances the tyrosine phosphatase activity as compared to Y564-site [17, 20, 21]. Thus, Src promotes SHP-1 phosphorylation that is crucial for the optimum activity of SHP-1 in the intact cells [23].

Src is a prototype of Src family kinases (SFKs) whose domain architecture and structural details are well-established [24–26]. Src autophosphorylation at Y416 residue of activation or A-loop is required for the functional kinase activity [27, 28]. Structure-function studies [29–31] and elucidation of 3-dimensional crystal structures of regulated SFKs [32, 33] reveal that phosphorylation and intramolecular interactions of both SH2 and SH3 domains play a role in the regulation of Src activity. Specifically, phosphorylation at the carboxy-proximal Y527 residue of Src, induced by a related tyrosine kinase Csk [31, 33, 34], leads to a ‘‘closed” or inactive conformation. Moreover, SH3, kinase domain, and the intervening linker region contribute to the negative regulation of Src [31].

In recent years, numerous studies have documented the molecular dynamics (MD)-based conformational readjustments in activated Src [35–38]; however, there has been no study addressing the comparative interaction paradigm of SHP-1 against active relative to inactive c-Src. Additionally, due to the lack of a C-terminal tail in the reported crystal structures of SHP-1 [19], it remains unclear how connections between the C-terminal tail of activated SHP-1 and c-Src establish dephosphorylation. Here, we ascribe the structural basis of c-Src (in both open and close transformation) association with activated SHP-1 through MD simulation assays. Through detailed binding analysis against active and inactive c-Src variants to SHP-1 (along with C-terminal tail), we propose that upon recognition of c-Src^open^, SHP-1 N-SH2 domain plays an inevitable role by moving away from the PTP domain and removing the SHP-1 C-terminal tail from the active site to access the Src. In contrast, parallel binding of c-Src^close^ C-terminal tail to WPD-loop and SHP-1 PTP domain mediates a stabilizing role in maintaining the inactive state of c-Src. We performed dynamic cross-correlation analysis to explore the inter-domain movement upon SHP-1 and Src binding in comparison to apo-SHP-1 and presented heatmaps, where a higher positive correlation increases pairwise movement, while a negative correlation means an inverse pairwise movement among residues. Subsequently, a custom Circos plot script (*https*:*//github*.*com/ponnhide/pyCircos**)* was used to elucidate the inter-domain movement higher than 70% positive correlation.

## Material and methods

### Data collection

Full length 3D structure of SHP-1 (Alphafold ID: AF-P29350-F1) was retrieved through *https*:*//alphafold*.*ebi*.*ac*.*uk/entry/P29350*. As C-terminal tail of this structure was disordered, SHP-1 C-terminal tail region (E533-K595) was predicted through Robetta (*https*:*//robetta*.*bakerlab*.*org/*) and joined to the Alphafold structure via USCF Chimera version 1.11 [39]. The resultant SHP-1 structure was phosphorylated at Y536 residue by UCSF Chimera 1.11 [39] and subjected to energy minimization through CHARMM-GUI input generator [40] of GROMACS 5.1.6 [41] using CHARMM36 force field [40, 41]. Subsequently, the minimized SHP-1 3D structure was validated by MolProbity [42], Verify3D [43], and MOE [44]. Finally, this structure was optimized by WinCoot [45]. In this study, the utilized SHP-1 structure is phosphorylated at Y536. The crystal structures of human c-Src in the open (PDB ID: 1Y57; resolution: 1.91Å) and close conformations (PDB ID: 2SRC; resolution: 1.50Å) were retrieved through RCSB PDB (*https*:*//www*.*rcsb*.*org/*). The activation loop (A-loop) of c-Src^open^ was phosphorylated at Y416 through UCSF Chimera 1.11 [39]. In contrast, the C-terminal tail of the c-Src^close^ exhibited Y527 phosphorylation.

### Molecular docking analysis

Receptor-ligand interactions play significant roles in various biological processes, and the knowledge of molecular associations may help in understanding numerous cellular pathways [46]. In this study, minimized SHP-1 was docked against c-Src^open^ and c-Src^close^ through the ClusPro [47], PatchDock [46] and an embedded refinement tool FireDock [48] to describe their interaction patterns. PatchDock accomplishes docking process through a segmentation algorithm based on the structure geometry. It recapitulates docking transformations that yield good complementary molecular shapes based on a small number of steric clashes and wide interface areas. PatchDock algorithm classifies the Connolly dot surface representation of the protein molecules as concave, convex, and flat patches [49]. The complementary patches are matched to generate the candidate transformations. A scoring function evaluates each candidate transformation, which considers both the atomic desolvation energy and geometric fit [50] to measure each candidate transformation. Finally, the most suitable candidate solution is selected among the redundant solutions based on RMSD (Root Mean Square Deviation) clustering. Overall, three major steps are followed in the PatchDock analysis: (i) surface patch matching, (ii) molecular shape representation, and (iii) filtering and scoring [51]. In order to confirm our docking results, SHP-1 binding to c-Src^open,^ and c-Src^close^ was evaluated by ClusPro [52]. ClusPro server (*https*:*//cluspro*.*org*) is a widely used protein-protein docking tool that performs docking in three steps: (1) rigid body docking, (2) RMSD-based clustering of the 1000 lowest energy structures, and (3) the removal of steric clashes by energy minimization.

### Molecular dynamics simulation assay

In order to gain further insight and evaluate dynamic behavior, conformational changes, and interaction stability, molecular dynamics (MD) simulation runs were performed for 300 ns. We used the CHARMM-GUI input generator [40] to build the systems for MD runs. All MD simulations were accomplished through GROMACS 5.1.6. [53] using CHARMM36 force field [40] and TIP3P water model [54]. All complexes were initially centered in the cubic periodic boxes having following dimensions: (SHP1: 12 x 12 x 12 nm, c-Src^open^: 12.5 x 12.5 x 12.5, c-Src^close^: 9.7 x 9.7 x 9.7, c-Src^open^-SHP1 complex: 14.2 x 14.2 x 14.2 nm, and c-Src^close^-SHP1: 14.7 x 14.7 x 14.7 nm). The distance between the solute and the edge of the water box was 10–15 nm. System neutralization was accomplished by adding appropriate numbers of Na^+^ and Cl^-^ counter-ions. MD simulation runs were executed under a constant pressure (1 atm) and temperature (303K) using a Nose-Hoover thermostat [55]. LINCS algorithm [56] was applied to constrain all bonds having hydrogen atoms with an integration time step of 2 fs. Long-range electrostatic interactions were estimated with a cut-off value of 1 nm for the direct interaction through fast, smooth Particle-Mesh Ewald (PME) summation [57]. Each system encountered a similar equilibration process under NVT [58] conditions and 1 fs integration time step covering 1000 ps MD simulations. An additional 1000 ps MD simulation under NPT [59] conditions with an isotropic Parrinello-Rahman barostat [60, 61] for 2 ps time period was performed to complete the equilibration stage. Finally, a 300 ns MD trajectory files were attained for each system. PDB files were generated for every 25 ns interval to evaluate and assess the system stability and structural changes. All MD trajectories were investigated through UCSF Chimera 1.11 and GROMACS tools. The behavior and stability of each system was scrutinized through GROMACS modules such as *g_rms*, *g_rmsf*, *g_hbond* and *g_covar* modules.

### Dynamic cross-correlation analysis

In an MD simulation environment, atoms are placed under classical mechanics constraints to characterize their behavior in terms of 3D coordinates. A dynamical system is needed to investigate how these Newtonian forces affect the atomic motions. In the dynamic cross-correlation method, neighboring atom movements are evaluated. Hence, dynamic correlation is a measure of atomic movements in a changing environment concerning other atoms. The dynamic correlation analysis was implemented using a Python script named as *calc_correlation*.*py* embedded in the MD-TASK module (*https*:*//github*.*com/RUBi-ZA/MD-TASK*). Input files were in the form of a trajectory (.xtc format) and topology (.gro format). A GROMACS library named as *mdtraj* library (*https*:*//mdtraj*.*org*/) gets input in the form of trajectory and topology files that are combined for the coordinate and atomic adjustments. Subsequently, NumPy (*https*:*//numpy*.*org/*) plugin was used to calculate the dynamic correlation values for the whole protein backbone under study. The vectorized NumPy assures hardware optimization, and values are calculated based on the following formula (Eq 1) [62].


$$C_{ij}=\frac{\langle \Delta r_{i}\cdot \Delta r_{j}\rangle }{\left(\sqrt{\langle \Delta r_{i}^{2}\rangle }\cdot \sqrt{\langle \Delta r_{j}^{2}\rangle }\right)}$$ (1)

Where Cij denotes the given correlation between the ith entity with respect to the jth one given the correlation values r for respective entities i and j [63]. The formula for each r is given as Eq 2:

$$r_{xy}=\frac{\sum (x_{i}-\bar{x})(y_{i}-\bar{y})}{\sqrt{\sum {\left(x_{i}-\bar{x}\right)}^{2}\sum {\left(y_{i}-\bar{y}\right)}^{2}}}$$ (2)

Where x and y are the two respective atoms for which the three-dimensional positions are negated from their mean position to understand the correlation. Value bounds for the dynamic correlation are the same as the canonical Pearson formula: +1 to -1, where +1 means perfect correlation, -1 means perfect anti-correlation, and 0 implies no correlation. After calculating the dynamic correlation, an NxN matrix was generated in a.txt format, where N designates the residue numbers in the system. Subsequently, correlation values were mapped in the form of a heatmap through an in-house made script named *plot_regions*.*py* which extends the MD-Task correlation script (*https*:*//github*.*com/RUBi-ZA/MD-TASK*). Here, the square matrix is given as input along with the start and end regions of the protein to be plotted as a heatmap. The script plots the whole protein in a.jpeg image format if no start and end regions are given. Files are submitted as input in the.txt format, and the output is generated using Matplotlib heatmap function (*https*:*//matplotlib*.*org/* *and* *https*:*//seaborn*.*pydata*.*org/*).

### Circos analysis

Originally, Circos was designed to comprehend the transcriptional regulation in the plant genome, where a plot is generated in a circle [64]. The idea had been repurposed here to visualize the dynamic correlation profiles of SHP-1 bound c-Src^open^ and c-Src^close^ structures through the simulation data. The script was developed in Python that works in the following manner.

Dynamic correlation values were assigned as input in a.txt format by marking individual domain start and ending regions. In the absence of these regions, the whole correlation matrix is considered. Additionally, a threshold is also used as an input parameter to isolate the regions with higher correlation values for plotting. Next, the script uses the G circle library (*https*:*//github*.*com/ponnhide/pyCircos**)* for drawing the individual domains. In the plot, the outer ring describes the region, the inner circle signifies the sequence, and the lines connecting the domains denote the correlation between the domains. The intra-correlation (within the domain) has not been addressed here, and the script only infers the inter-domain correlation. The output is in the.pdf format, converted into.jpeg or.png format.

## Results

### Structural evaluation for c-Src^open,^ c-Src^close^ and SHP1

**Fig 1:**
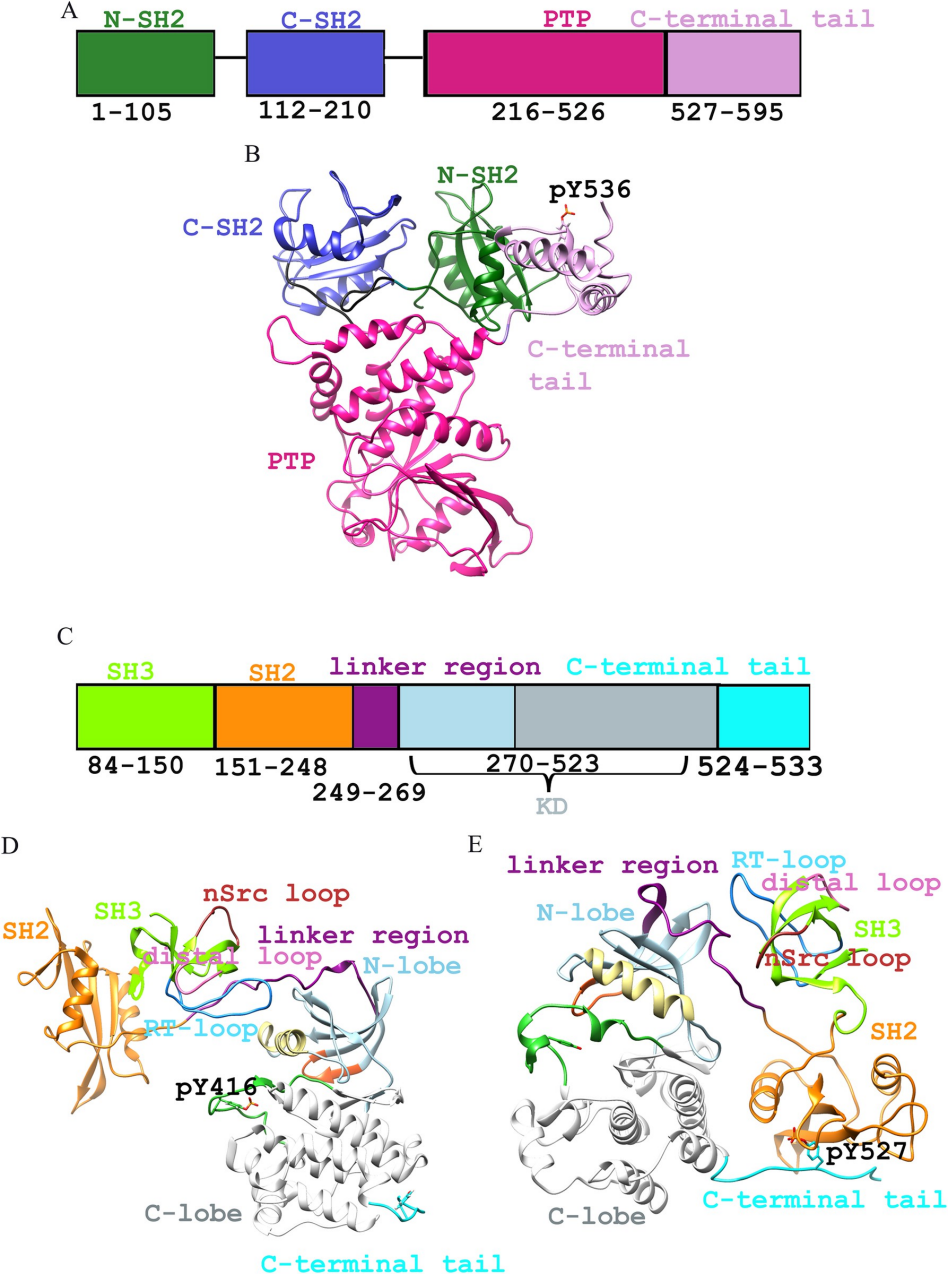
3D structures of SHP-1, c-Src^open,^ and c-Src^close^. (A) Schematic and (B) 3D structure representation of SHP-1. (C) Schematic and (D and E) 3D representation of c-Src open and close conformation. SHP-1: At the N-terminus, two tandems of SHP-1 are shown in dark cyan (N-SH2) and deep blue (C-SH2) color, while PTP is in pink and C-terminal tail is indicated in plum color. Color codes for SH3, SH2 domain, and linker region are displayed in chartreuse, orange, and dark magenta colors, respectively. Kinase domain, N-lobe, C-lobe, and A-loop are shown in light blue, white, and lime green colors, respectively. Gly-loop and C-terminal tail are shown in orange-red and cyan colors, while phosphorylated residues (pY416 and pY527) are shown in the deep pink sphere, respectively.

Human SHP-1 comprises two SH2 domains at the N-terminal regions, followed by a catalytic domain (PTP domain) and a C-terminal tail (Fig 1A and 1B) [7, 9]. Human c-Src is comprised of SH3 (84-145aa) and SH2 (151-248aa) domains, a linker region (249-269aa), a kinase domain (270-523aa), and a C-terminal tail (524-533aa), respectively (residue numbers were referred corresponding to chicken c-Src) (Fig 1C–1E) [65, 66]. The comparison of SHP-1 structure with the Alphafold structure has been included in S1 Fig and an RMSD value of 0.428 angstroms was obtained, indicating that these structures are exactly similar. Ramachandran scores for c-Src^open^ (98.00%), c-Src^close^ (93.71%), and SHP1 (94.44%) structures suggested that significant numbers of residues were lying in the sterically allowed regions. The Ramachandran plot combines the four separate Ramachandran maps (as shown to the right) using shapes to distinguish the membership to a particular class (General, Proline, Glycine, and Pre-Proline). Within this scheme, Glycines are shown as diamonds, Prolines as triangles, residues preceding Prolines (pre-Proline) have a rectangular shape, otherwise (General case) they are drawn as small squares (S1 Table in S1 File; S2 Fig). Through Verify3D analysis, average 3D-1D scores for c-Src^open^, c-Src^close^, and SHP1 were 97.34%, 97.10%, and 90.76%, respectively (S3 Fig).

### Molecular docking analysis of c-Src^open^ and c-Src^close^ against SHP-1

The molecular docking runs were performed to determine the SHP-1 binding region required for c-Src^open^ (SHP-1 activation by phosphorylation at Y536) and c-Src^close^ (Y527 dephosphorylation of c-Src^close^). In the c-Src^open^-SHP-1 complex, binding of both SH3 and SH2 domains of c-Src was observed with two tandems (SH2) of SHP-1 (S4A and S4B Fig), while in the c-Src^close^-SHP-1 complex, binding of C-terminal tail of c-Src^close^ was evident to the PTP domain of SHP-1 (S4C and S4D Fig). PatchDock specific energy values for c-Src^open^-SHP-1 complex and c-Src^close^-SHP-1 complex were -15.28 and -20.06 kcal/mol, respectively. The details of hydrogen bonds, non-bonded, and salt bridge interactions for c-Src^open^-SHP1 and c-Src^close^-SHP1 complexes are listed in the S2 Table in S1 File.

The PatchDock results were further validated through another independent tool ClusPro.. The statistical scores obtained by the ClusPro server for the optimal clusters of c-Src^open^-SHP-1 and c-Src^close^-SHP-1 complexes are illustrated in the S5 Fig and S2 Table in S1 File.

### MD simulation analysis

**Fig 2:**
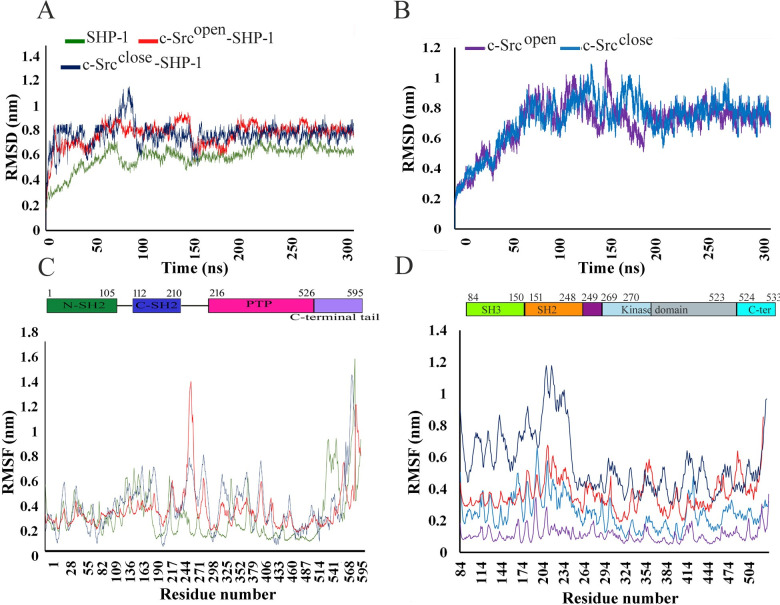
Time-dependent analysis. (A) RMSD plots of apo-SHP-1, c-Src^open^-SHP-1, and c-Src^close^-SHP-1 complexes, (B) RMSD plot of apo-c-Src^open^ and apo-c-Src^close^. (C) RMSF plots over 300 ns for apo-SHP-1 and its bound forms with c-Src^open^ and c-Src^close^. (D) RMSF plots for apo-c-Src^open^ and c-Src^close^ and its complexes with SHP-1. Apo-SHP-1 (green), c-Src^open^-SHP-1 (red), c-Src^close^-SHP-1 (blue), c-Src^open^ (purple), and c-Src^close^ (light blue).

To validate the findings of MD simulation runs, we performed three replicas of 300 ns simulations for apo-SHP-1 and its complexes with c-Src^open^, c-Src^close^, apo c-Src^open^, and c-Src^close^ to achieve the coverage of energetic and conformational space (S6 Fig). Subsequently, the average values were computed from these replicas and plotted. RMSD profile analysis exhibited true structural convergence for all systems (Fig 2A and 2B). Overall, apo-SHP-1 and c-Src^close^-SHP-1 complex attained stability at 150 ns and demonstrated 0.2 nm and 0.8 nm RMSD values, respectively, while c-Src^open^-SHP-1 complex achieved stability at 200 ns time scale (Fig 2A). c-Src^open^ and c-Src^close^ attained stability at 200 ns and demonstrated 0.7 nm to 0.75 nm RMSD values, respectively (Fig 2B).

**Fig 3:**
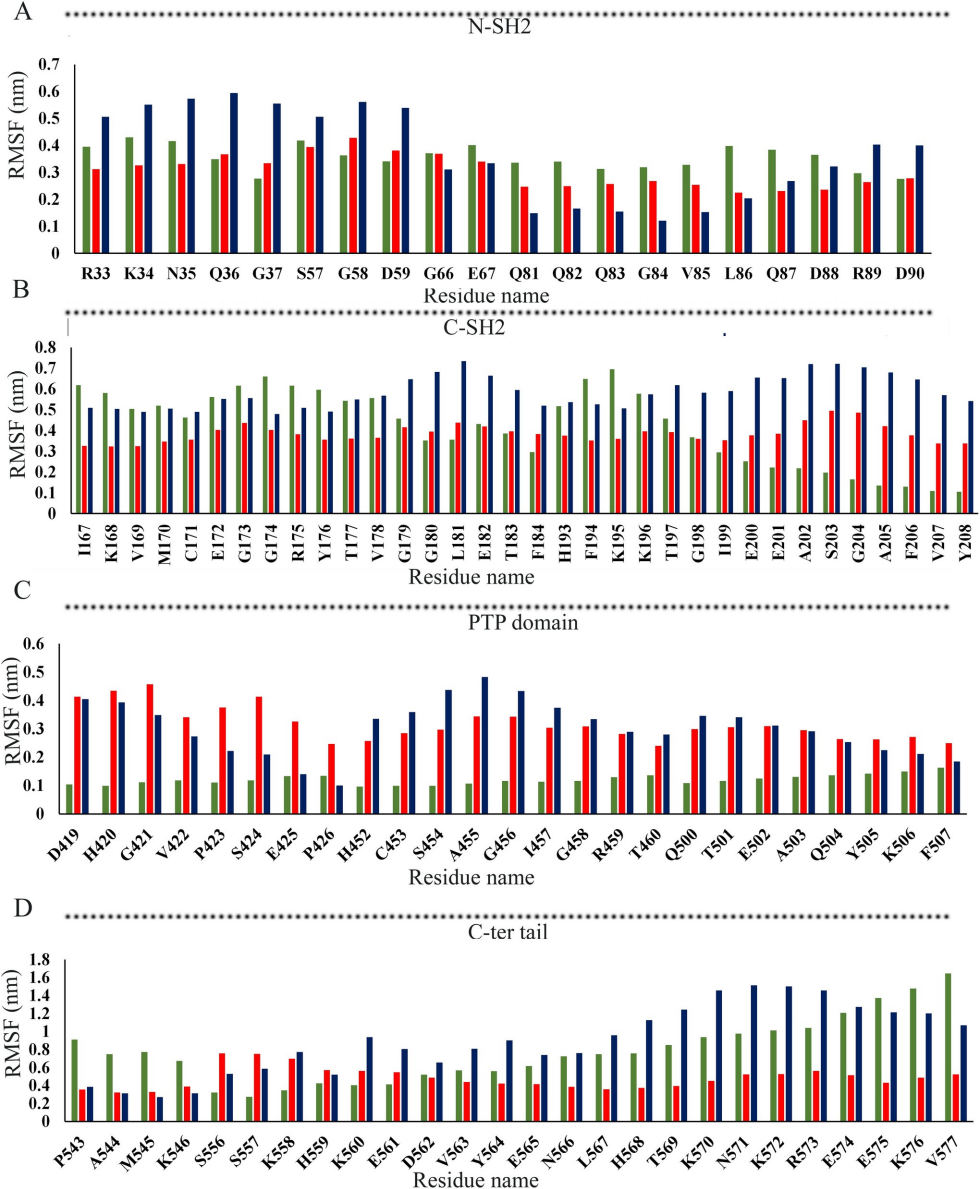
Comparative RMSF bar plots for SHP-1 domains. (A) N-SH2, (B) C-SH2, (C) PTP, and (D) C-terminal tail. Detailed color codes are given in the Fig 2 legend.

RMSF analysis was performed to evaluate the conformational changes in SHP-1, c-Src^open^-SHP-1, c-Src^close^-SHP-1 complexes, (apo) c-Src^open^ and c-Src^close^ (Fig 2C and 2D). In c-Src^open^ and c-Src^close^, major fluctuations were observed in T84-F150 (SH2 domain) and G151-S248 (SH3 domain), ranging from 0.7–0.75 nm, respectively (Fig 2D). In the N-SH2 domain of c-Src^close^-SHP-1 complex and apo-SHP-1, predominant fluctuations were observed in R33-G37 (0.20–0.59 nm), S57-D59 (0.38–0.53 nm), G66-E67 (0.31–0.40 nm), and Q81-D90 (0.24–0.40nm) regions, while the c-Src^open^-SHP-1 complex was relatively stable throughout simulation run (Fig 3A). Furthermore, in the C-SH2 domain (S116-G159), pronounced fluctuations were detected in I167-F184 (0.32–0.73 nm), and H193-Y208 (0.35–0.71 nm) regions, while the C-SH2 domain remained stable in the c-Src^open^-SHP-1 complex (Fig 3B). In the c-Src^close^-SHP-1 complex, PTP signature motif (H/V)CX5R(S/T), WPD loop motif (WPDXGXP), and Q loop motif (QTXXQYXF) exhibited more transitions (Fig 3C). Overall, more fluctuations were witnessed in the PTP domain of SHP-1 in the c-Src^close^-SHP-1 complex. D543-K546 (0.35–1.91 nm) and S556-V577 (0.39–1.64) exhibited major fluctuations in the C-terminal tail (Fig 3D).

To examine the H-bonds, non-bonded contacts, and salt bridges, dynamic trajectories of c-Src^open^-SHP-1 and c-Src^close^-SHP-1 complexes were used to generate complexes at regular time intervals of 25, 50, 75, 100, 125, 150, 175,200, 225, 250,275 and 300 ns. In contrast to c-Src^close^-SHP-1, c-Src^open^-SHP-1 demonstrated more H-bonds (S3 Table in S1 File) throughout the simulation run. For both Src-open and close structures, convergence was achieved at 20 ns, while the SHP-1 structure converged at about 60 ns. Through comparing the RMSD values using the initial structures, the stationary trajectory state revealed a deviation of 8Å from the starting structure unit 80 ns for Src structures; thus, a structure obtained at 90 ns was used as reference structure. For SHP-1, the structure obtained at 100 ns was used as a reference (S7 Fig).

### Dynamic correlation and Circos analysis

**Fig 4:**
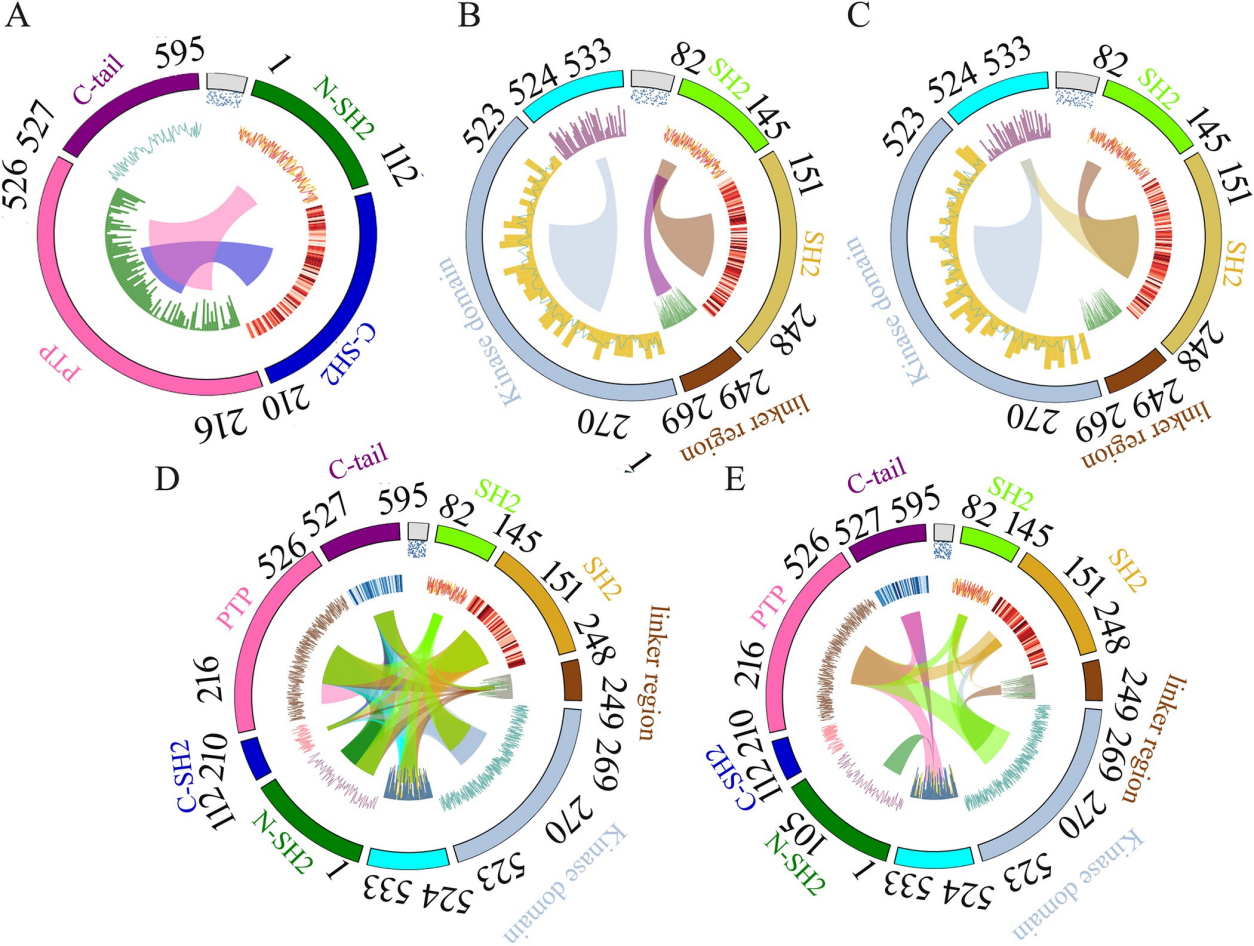
Circos plots with >70% cross-correlation value. (A) SHP-1(B) c-Src^open^, (C) c-Src^close^, (D) c-Src^open^- SHP-1, and (E) c-Src^close^-SHP-1. Src domains are represented in different colors: SH3 domain (light green), SH2 domain (orange), linker (brown), kinase domain (gray), and C-terminal tail (cyan). SHP-1 domains are indicated as follows: N-SH2 (green), C-SH2 (blue), PTP (pink), and C-terminal tail (purple).

To understand the movement of the protein under study, this study used dynamic cross-correlation technique. Dynamic correlation implicated the combinatorial atomic movement. Correlation in the positive 1 direction showed that the atoms were positively correlated. The reverse was true for the negative 1 direction, where 0 meant no correlation. These values were calculated in a highly dynamic environment through the molecular dynamic simulation setting. Circos plots were used to visualize the domains with a >0.7 or 70% cross-correlation value. Correlation heatmaps provide the correlation landscape of residues lying inside the protein in a pairwise manner. Circos plots were introduced to highlight the inter-domain movement. In a Circos plot, the protein box is represented as the outermost circle that is located at the top with a grey box and moves clockwise along the sequence length. The outbound circle represents the domains. The inbound one indicates sequence, and the line bars that flow from one domain to the other represent correlations higher than or equal to 70%. The intra-domain correlation was omitted here to avoid cluttering in the plots since the inter-domain regions were taken into consideration. Besides this rationale, each domain needs to be correlated to evaluate the collective residue movement in a given domain. In SHP-1, N-SH2 and C-SH2 domains were highly correlated with the PTP domain. Interestingly, anti-correlation was observed between C-terminal and N-SH2 domain of SHP-1 (Fig 4A and S8A Fig). In the case of c-Src^open^, there was a sparse inter-domain correlation. SH2 domain was positively correlated with the SH3 and linker region. Evidently, Circos plot revealed a positive correlation among domains. A more negative correlation was observed between the SH2, kinase domain and the C-terminal tail. SH3 domain also showed an anti-correlation pattern between the linker and subsequent domain (Fig 4B and S8 Fig). In case of c-Src^close^, the SH2 domain was positively correlated with SH3, while the SH3 domain was positively correlated with the C-terminal tail. The C-terminal region was also positively correlated with the kinase domain. A negative correlation was witnessed between the kinase domain and SH2 and SH3 domains (Fig 4C and S8 Fig). In the c-Src^open^-SHP-1 complex, the correlation heatmap and Circos plots demonstrated intense correlation in the whole landscape (Fig 4D and S8D Fig). Interesting correlations could be seen in the c-Src^close^ coupled with SHP-1 (Fig 4E and S8E Fig). At the intra-domain level, SH3, SH2, and the linker region of c-Src possessed positive correlations with KD, while the linker region was correlated with the C-terminal region. In the case of SHP-1, the PTP domain exhibited a positive correlation with SHP-1 C-terminus. The intermolecular interaction analysis revealed regions with a positive correlation for c-Src^close^-specific SH3, SH2, and C-terminal tail with the SHP-1 PTP domain. Additionally, the c-Src^close^ C-terminal tail demonstrated a positive correlation with the SHP-1 N-SH2 and C-terminal tail (Fig 4E and S8E Fig).

## Discussion

SHP-1 is a cytosolic tyrosine phosphatase that is primarily expressed in the hematopoietic cells and controls many cellular functions to control the flow of information from the cell membrane to the nucleus [11–14]. It acts as a negative regulator in numerous signaling pathways [67]. Recently, it has been reported that c-Src associates with the SH2 domain of SHP-1 in both platelets and lymphocytes cells [19] and phosphorylates at Y536 residue of SHP-1 C-terminal tail leading to enhanced activity of phosphatase [19, 22]. Subsequent dephosphorylation of c-Src pY527 by SHP-1 results in the activation of Src tyrosine kinase [21]. Although the role of SHP-1 in maintaining the overall phosphorylation status and activity of c-Src is well known, their binding characteristics and the key residue involvements in the activity paradigm are largely unknown.

**Fig 5:**
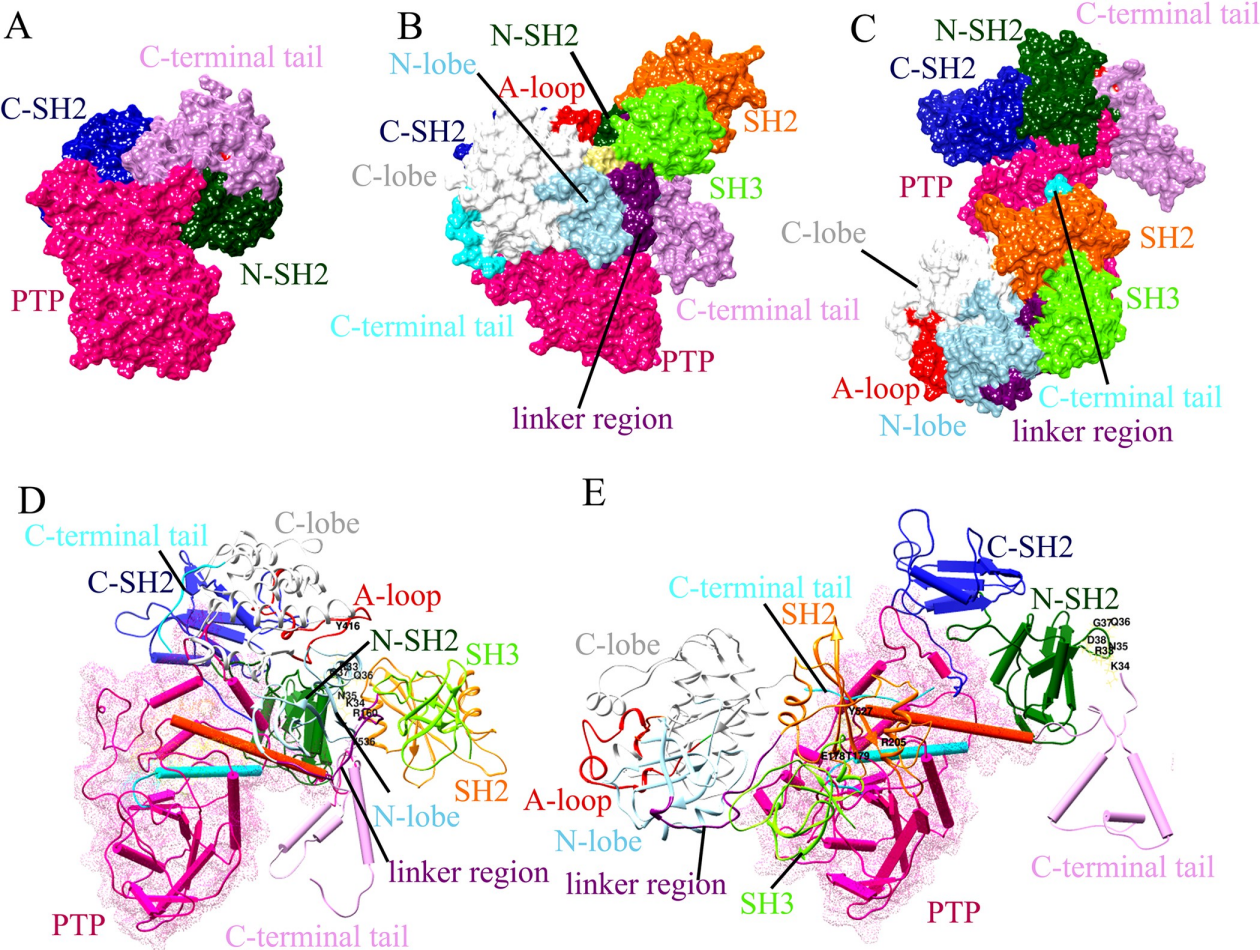
Surface view and the conformational switches in SHP-1 upon binding of c-Src^open^ and c-Src^close^. Surface view representation of (A) SHP-1, (B) c-Src^open^-SHP-1 complex, and (C) c-Src^close^-SHP-1 complex. Conformational switches in (D) c-Src^open^-SHP-1 complex and (E) c-Src^close^-SHP-1 complex. The corresponding text indicates the color codes of individual substructures.

Current study characterizes the binding patterns of c-Src^open^ (PTR416) and c-Src^close^ (PTR527) with SHP-1 (PTR536) through *in silico* approaches. Convincingly, in case of c-Src^open^, MD simulation analysis revealed the participation of both SH2 and SH3 domains in binding to the SHP-1 N-SH2 domain. In contrast, an active role of c-Src^close^ C-terminal tail was observed in the interaction with SHP-1 PTP domain. Intriguingly, more conformational readjustments were witnessed in c-Src^open^ upon binding to SHP-1 (Fig 5). SHP-1-specific _33_-RKNQG-_37_ motif (N-SH2) association with SH3 (D117) and SH2 (R160, A165, and E166) domains of c-Src^open^ induces the recognition-induced conformational changes in the SH2 domain for binding to SHP-1 C-terminal tail (Fig 5). Consequently, R160 residue pairs with N35 and Y536 residues of SHP1 through hydrogen bonding and associates with SHP-1 E535 side chain through a salt bridge (S3 Table in S1 File). These findings are in good agreement with the previous studies where both SH3 and SH2 domains cooperatively regulate c-Src^open^ activity through a physical interaction [68]. As compared to apo-SHP1, N-SH2 domain moves opposite to the C-terminal tail upon binding to c-Src^open^. Evidently, apo-SHP1 C-tail covers the active site of the PTP domain and upon c-Src recognition, N-SH2 domain pushes it aside to make the active site more accessible (Fig 5). Upon substrate binding, N-SH2 domain moves away from the PTP active site, as reported for the phosphatase-active form of SHP-1/2 [7, 9], while in the corresponding inactive form, this domain keeps SHP protein in an auto-inhibitory state through blocking the active site. Mechanistically, the reported SHP-1 and SHP-2 crystal structures lack C-tails, and SHP-1 N-SH2 D’E loop enters into the active site of PTP domain and prevents the substrate binding to phosphatase [7, 9]. In this study, through detailed binding analysis of active versus inactive c-Src to SHP-1 (including C-terminal tail), we anticipate that cooperative binding of both SH2 domains and the C-terminal tail of SHP-1 to SH2 and KD of Src^open^ can lock the Src open conformation. SHP-1 attains a more open conformation upon binding to c-Src^open^ that is supported by the proportional increase of SHP-1 surface volume. Interface surface areas of SHP-1, c-Src^open^-SHP-1 and c-Src^close^-SHP-1 complex were 29.59e3, 55.60e3 and 50.34e3, whereas volumes were 77.46e3, 135.7e3 and 137.4e3, respectively (Fig 5). Interestingly, attachment of K298 residue of c-Src^open^ KD through hydrogen bonding to SHP-1 E224 (PTP domain) and E517 (C-terminal tail) indicates its potential role in stabilizing c-Src^open^ conformation. K298 residue has been reported to regulate c-Src global kinase activity, while K298E mutation results in c-Src closed conformation [69].

SHP1-dependent c-Src activation through pY527 dephosphorylation [70] relies on the conformational change in the inhibitory C-terminal tail that eases the recruitment of the SH2 domain to phosphotyrosine residue. As a result, inhibition of the phosphatase domain is lifted by the N-SH2 domain of SHP-1. Similar observations have been reported for Lyn-induced phosphorylation of SHP-1 Y536 residue that mediates a conformational change to recruit SHP-1 through its SH2 domains to the phosphotyrosine residue leading to the activation of phosphatase inhibitory potential [71]. Another report suggests binding of SHP2 (a close homolog of SHP1) to c-Src to dephosphorylate it at Y527/Y530 [72]; however, a phosphatase-inactive mutant of SHP-2 is also capable of c-Src activation, suggesting that c-Src activity may not solely be regulated by the dephosphorylation process [73].

In the case of the c-Src^close^-SHP-1 complex, significant contributions of WPD-loop (WPDXGXP) and Q-loop (QTXXQYXF) of the SHP-1 PTP domain were observed in the transition of close-to-open conformation. The binding preferences for c-Src^close^ C-terminal tail residues (T523, E524, Q526, pY527, and N532) to WPD-loop D419 and SHP-1 PTP domain (K232, K356, H420, V422, and S424) play a critical role in promoting the dephosphorylation activity and activation of c-Src (S3 Table in S1 File). These findings are in good agreement with our dynamic correlation analysis, where c-Src^close^ C-terminal tail (524–533 aa) is positively correlated with SHP-1 PTP domain and KD, and movement of SH2-SH3 region is highly correlated to that of SHP-1 C-SH2 domain, suggesting their cooperative role in promoting the c-Src closed conformation (Fig 4).

Collectively, our findings delineate the comparative analysis of SHP-1 binding to both active and inactive forms of c-Src to uncover the underlying global conformational switches that govern the state transition basis of Src due to individual movements of regulatory domains. Our study will expand the previous knowledge of SHP-1-dependent tyrosine dephosphorylation and add significant information regarding the kinase activation to curb cancer development. In the case of the c-Src^close^-SHP-1 complex, significant contributions of WPD-loop (WPDXGXP) and Q-loop (QTXXQYXF) of the SHP-1 PTP domain were observed in the transition of close-to-open conformation. Conversely, in case of c-Src^open^, both SH2 and SH3 domains are responsible in binding to the SHP-1 N-SH2 domain.

## Supporting information

S1 File (DOCX)

S1 Fig Comparsion of modeled SHP-1 with Alphafold structure. (TIF)

S2 Fig The Ramachandran plot displays the phi-psi torsion angles for (A) c-Src^open^, (B) c-Src^close^ and (C) SHP-1.
(TIF)

S3 Fig Verify3D plots for (A) c-Src^open^, (B) c-Src^close,^ and (C) SHP-1. Y-axis demonstrates the Verify3D score for each residue, whereas the X-axis represents the residue number.
(TIF)

S4 Fig Interaction analysis of SHP-1 versus c-Src^open^ and c-Src^close^. (A and B) c-Src^open^-SHP-1. (C and D) c-Src^close^-SHP-1. SHP-1: N-SH2 in deep green, C-SH2 in deep blue, PTP in pink and C-terminal tail in plum. Open and close conformation of c-Src. Color code: SH3, SH2 domain, and linker region are displayed in chartreuse, orange, and dark magenta colors. Kinase domain, N-lobe, C-lobe, and A-loop are shown in light blue, white, and lime green colors. Gly-loop and C-terminal tail are shown in orange-red and cyan colors, while phosphorylated residues (pY416 and pY527) are shown in the deep pink sphere, respectively.
(TIF)

S5 Fig ClusPro interaction analysis of SHP-1 versus c-Src^open^ and c-Src^close^. (A and B) c-Src^open^-SHP-1. (C and D) c-Src^close^-SHP-1. SHP-1: N-SH2 in deep green, C-SH2 in deep blue, PTP in pink and C-terminal tail in plum. Open and close conformation of c-Src. Color code: SH3, SH2 domain, and linker region are displayed in chartreuse, orange, and dark magenta colors. Kinase domain, N-lobe, C-lobe, and A-loop are shown in light blue, white, and lime green colors. Gly-loop and C-terminal tail are shown in orange-red and cyan colors, while phosphorylated residues (pY416 and pY527) are shown in the stick, respectively.
(TIF)

S6 Fig RMSD plots. Comparative RMSD for (A) apo-SHP-1 (green), (B) c-Src^open^-SHP-1 (red), (C) c-Src^close^-SHP-1 (blue), (D) c-Src^open^ (purple), and (E) c-Src^close^ (sky blue), and their replicas are represented in deep pink and orange colors, respectively.
(TIF)

S7 Fig Comparison of the 90 ns and 300 ns structure of c-Src^open^, c-Src^close^, and 100 ns and 300 ns structures of SHP-1. The 90 ns for c-Src^open^, c-Src^close^, and SHP-1 (100ns) are green, while 300 ns are orange. (A) **c**-Src^open^ (B) **c**-Src^close^ (C) SHP1. RMSD values are labeled in angstrom.
(TIF)

S8 Fig Dynamic correlation heatmap plots to estimate positive and negative correlated motions. (A) SHP-1, (B) c-Src^open^, (C) c-Src^close^, (D) c-Src^open^- SHP-1, and (E) c-Src^close^-SHP-1 plots.
(TIF)
