# Supplementary material for: SHP-1 tyrosine phosphatase binding to c-Src kinase phosphor-dependent conformations: A comparative structural framework
Source: PLoS One. 2023 Jan 13;18(1):e0278448. doi: 10.1371/journal.pone.0278448 (PMC9838854; doi:10.1371/journal.pone.0278448)
Supplement: S1 File — (DOCX) [file pone.0278448.s001.docx]

**S1 Table.**

| **Tools**  **Proteins** | **MolProbity** | **Prosa-Web** | **Verify3D** |
| --- | --- | --- | --- |
|  | **Ramachandran favored** | **Z-score** | **3D-1D score** |
| c-Src^open^ | 98.00% | -10.8 | 97.34% |
| c-Src^close^ | 93.71% | -10.81 | 97.10% |
| SHP1 | 94.44% | -9.89 | 90.76% |

**S2 Table**

| **PatchDock results** | |
| --- | --- |
| **c-Src^open^-SHP-1 complex** | **c-Src^close^-SHP-1 complex** |
| **H-bonds** | |
| C277:D185 | T180:R352, R205: S416, T521:E502, T523: K356, T523:D419 andT523:H420 |
| **Non-bonded contacts** | |
| **T154**:E535, **R156**:Y536, **R156**:K34, **E157**:L12, **R160**:**R33**, **R160:L12,** **N164**: R 33, **E178**:E535, C277:S186, C277:D185, F278:D185, Q420:G73, Q420:E172, G421:R175, F424:T183, R469:L181, R469:E182, R 469: L181, R469:E182 | T179:R352, T180:R352, K181:V354, R205:S416, R205:R352, R205:L415, D208:D386, Y213:T388, T521:E502, T521:F248, T523:K356, T523:D419, T523:H420, P525:D419, P525:P418, P525:D419, P525:H420, Q526:P418, Y527:S424 and P529:E425 |
| **Salt bridges** | |
| R469:E182 | Nil |
| **ClusPro2.0 results** | |
| **c-Src^open^-SHP-1 complex**  (**Weighted Score (**lowest energy): -966.6) | **c-Src^close^-SHP-1 complex**  (**Weighted Score (**lowest energy): -819.8) |
| **H-bonds** | |
| K298:E108 | E178:K356, R205:L415, R205:S416, E510:R358, T521:E502, E524:K356 Y527:S424 |
| **Non-bonded contacts** | |
| M82:K589, R156:K34, **R156:R89**, **R156:Y536**, **L163:K34**, C277:S186, F278:D185, K298:E108, Q420:G173, G421:R175, F424:T183 and R469:L181 | E178:K356, T179:E355, R205:L415, R205:S416, Y213:T388, S216:T388, E510:R358, T521:E502, T521:F248, T521:T501, T523:H420, E524:K356, Y527:S424, P529:S424 andP529:E425 |
| **Salt bridges** | |
| **R160:E15**, K298:E108 | E510:R358, E524:K356 |

**S3 Table**

| **PBD** | **c-Src^open^:SHP-1** | **c-Src^close^: SHP-1** |
| --- | --- | --- |
| **Hydrogen bonds** | | |
| 25 ns | E115:K576  **D117:K572**  **R160:E535**  **K181:E586**  K298:E224  K298:E517  E332:K521  R469:E182  R469:T183 | E510:R358  Q526:V422 |
| **Non-bonded contacts** | | |
|  | E115:K576  D117:K572  D117:E575  **K152:E586**  **R156:R89**  **R156:Y536**  **R160:E535**  **R160:Y536**  **R160:E535**  **R160:Y536**  **R160:R53**  **E166:K34**  **K181:E586**  K298:E517  K298:E224  K298:E517  K298:E224  K298:E517  P299:K521  E332:K521  E332:E524  R469:E182  R469:T183  R469:E182  R469:T183  R469:F184  R469:F184  R469:E182  R469:T183 | T179:R352  A194:D238  E510:R358  T523:H420  E524:K356  E524:D419  Q526 :A503  Q526 :V 422  Q526 :V422  Q526:G421  Q526:V422  L533:K232 |
| **Salt bridges** | | |
|  | E115:K576  D117:K572  **K152:E586**  **R160:E535**  **E166:K34**  **K181:E586**  K298:E224  K298:E517  E332:K521 | E510:R358  E524:K356 |
| 50 ns | **Hydrogen bonds** | |
|  | D17:K576  **R160:E535**  **R160:E535**  R469:T183 | K181:T388  K205:E 425  R217:E224  E517:K356  **E524:K356**  **Q526:V422** |
| **Non-bonded contacts** | | |
|  | E115:K579  D117:K576  **R160:E535**  **R160:E535**  **R160:Y536**  **T252:K572**  P299:K521  R332:R573  R332:E524  L435:R175  R469:T183  R469:F184  R469:T183  R469:D185  R469:T183  D473:E182 | T179:R352  T180:T388  K181:T388  R205:S424  R205:E425  R217:E224  E510:R358  Y511:R358  E517:K356  **E524:K356**  **Q526:G421**  **Q526:V422**  **Q526:A503**  **Y527:S424**  **P529:S424**  **N532:K232**  **L533:L228**  **L533:N231** |
| **Salt bridges** | | |
|  | E115:K579  D117:K576  **R160:E535**  E332:R573  R469:D185 | R205:E425  R217:E224  E517:K356  **E524:K356** |
| 75 ns | **Hydrogen bonds** | |
|  | D117:K579  **R160:E535**  G300:E524  E331:K576  E332:K521  E470:K196 | T180:S416  R205:E425  E517:R358  **E524:D419**  **Q526:V422**  **Y527:S424** |
| **Non-bonded contacts** | | |
|  | D117:K579  **R156:Y536**  **R160:E535**  **R160:Y536**  **N164:K34**  **Q251:K570**  P299:E524  P299:K521  G300:K520  E331:K576  E332:K521  E332:E524  V467:K196  N468:D189  R469:F184  R469:F184  E470:K196  E470:K196 | T179:R352  T179:L415  T179:S416  T180: S416  K 181: T388  K181:D386  K181:T387  K181:T388  K181:L415  K181:D386  K181: T387  R205:E425  E510:R358  E510:G357  A514:R358  E517:K356  E517:K356  E517:R358  **E524:P418**  **Q524:D419**  **Q526:S424**  **Q526:P423**  **Q 526: V22**  **Q526:P423**  **Y527:S424**  **P529: N231**  **L533: K232**  **L533: L228**  **L533: N231** |
|  | **Salt bridges** | |
|  | D117:K579  **R160:E535**  E331:K576  E332:K521  E470:K196 | K181:D386  R205:E425  E517:K356  E517:R358 |
| 100 ns | **Hydrogen bonds** | |
|  | E115:K579  G116:K579  D117:K576  D117:K579  **R156:E535**  **R160:E535**  K298:E224  G465:K196  N468:D189  R469:T183 | T180:D386  R205:E425  R205:E425  **E524:V422** |
| **Non-bonded contacts** | | |
|  | E115:K579  G116:K579  D117:K579  **R156:E535**  **R156:E535**  **R156:Y536**  **R160:E535**  **R160:K34**  **R160:E535**  **R160:Y536**  K298:E517  K298:E224  K298:E517  P299:K521  E332:K521  G465:K196  V467:E192  V467:K196  R469:T183 | T179:L415  T180:D386  G182:T388  A183:T388  R205:E425  Y511:G357  Y511:R358  A514:R358  E524:G421  E524:V422  P525:P418 |
|  | **Salt bridges** |  |
|  | D117:K576  D117:K579  **R156:E535**  **R160:E535**  K298:E224  K298:E517  E332:K521  E470:K196 | R205:E425 |
| 125 ns | **Hydrogen bonds** | |
|  | **R160:E535**  **E166:R33**  K298:E224  K298:E517  P299:K520  E332:K521  E470:K196 | E178:R352  T180:D386  R205:E425  D518:R358  D518:R358 |
| **Non-bonded contacts** | | |
|  | **R156:Y536**  **R160:E535**  **R160: Y536**  **R160 K34**  **L163:Q36**  **L163:K34**  **E166:R33**  **K195:Q36**  **G196:Q36**  K298:E517  K298:E224  K298:E517  P299:K520  P299:E517  P299:K520  P299:E517  E332:K521  V467:E192  E470:K196 | E178:R352  T79:L415  T179:T388  T179:S 416  T180:T388  R205:E425  E510:G357  A514:R358  F515:R358  D518:R358  **P525:P418**  **L533:L228** |
|  | **Salt bridges** |  |
|  | R160:E535  E166:R33  K298:E224  K298:E517  E332:K521  E470:K196 | R205:E425  D518:R358 |
| **Hydrogen bonds** | | |
| 150ns | **D117: K572**  **R160:N35**  **L163:Q36**  **A165: R33**  **Q251:K572**  **Q251:N571**  K298:E224  K298:E517  G300:E524  T301:K520  E332:K521  E470:K196 | T180:D386  R205:E389  R205:E425  R205:E425  R217:E224 |
|  | **Non-bonded contacts** | |
|  | D117:K572  D117:K576  **R156: Y536**  **R156:R89**  **R156:Y536**  **R160:Y536**  **R160:K34**  **AR160:N35**  **R160:R53**  **A160: Y536**  **L163:Q36**  **N164:R33**  **N164:K34**  **A165:R33**  **GE166:R33**  **L197:Q36**  Q251:N571  Q251:K572  Q251:K570  Q251:N571  K298:E224  K298:E224  K298:E517  K298:E224  K298:E517  P299:E524  P299:K520  P299:E517  G300:E524  T301:K520  E332:K521  E332:E524  E332:K521  Q420:D185  V467:K196  N468:D189  N468:D189  E470: K196 | T180:D386  R205:E389  R205:E425  R217:E224  Y511:R358  L514:R358  **E524:D419** |
|  | **Salt bridges** | |
|  | D117 K572  D117 K576  K298 E224  K298 E517  E332:K521  E470:K196 | R205:E389  R205:E425  **E524:K356** |
|  | **Hydrogen bonds** |  |
| 175ns | D117:K576  R160:N35  R160:E535  A165:R33  K272:E425  E280:K521  K298:E224  K298:E517  Y300:E524  K351:D238  N468:E192  N468 :D189  E470:K196 | E178:R352  K195:E237  K195:D238  R205:E389  R205:E425  R205:E425  R379:G357  E510:R358  **E524:K506**  **Q526:V422**  **Y527:S424** |
|  | **Non-bonded contacts** |  |
|  | D117:K572  D117: K576  D117: E575  **R156:Y536**  **R156:R89**  **R160:E535**  **R160:N35**  **R160:E535**  **R160:Y536**  **R160:K34**  **R160:N35**  **R160:E535**  **L163:Q36**  **L163:Q36**  **N164:K34**  **N164:R33**  **A165:R33**  **A165:Q36**  **A165:R33**  L197:Q36  Q251:K570  K272:E425  Q275:E517  E280:K521  E280:E517  K298:E517  K298:E224  K298:E517  P299:E524  P299:K521  P299:E517  G300:E524  E332:R573  K351:D238  K351:D238  R460:E237  V461:S236  N468:E192  N468:D189  N468:E192  N468:D189  N468:D189  R469:D189  E470:K196 | E178:R352  T179:L415  T179:L415  T180:T388  K195:D238  K195:E237  K195:D238  R205:E389  R205:E425  R205:E389  R205:E425  Y213:T388  R379:G357  E510:R358  **E524:K506**  **E524:K506**  **P525:G421**  **Q526:S424**  **Q526:P423**  **Q526:S424**  **Y527:S424** |
|  | **Salt bridges** | |
|  | D117:K572  D117:K576  R160:E535  K272:E425  E280:K521  K298:E224  K298:E517  K351:D238  E470:K196 | E178:R352  K195:D238  R205:E389  R205:E425  E510:R358  **E524:K506** |
|  | **Hydrogen bonds** |  |
| 200ns | E115:K579  D117:K576  D117:K572  S134:K572  **R160:N35**  **R160:K34**  **R160:N35**  K272:E425  E280:K521  K298:E517  G300:E524  K351:D238  N468:D189  E470:K196 | E178:R352  R205:E389  R205:E425  R205:E425  E517:R358  E524:K356  Q526:V422 |
|  | **Non-bonded contacts** |  |
|  | E115:K579  D117:K572  D117:K576  S134:K572  N135:K572  **R160:Y536**  **R160:N35**  **R160:R53**  **R160:Y536**  **R160:K34**  **R160:N35**  **A165:Q36**  K272:E425  E280:K521  K298:E517  K298:E224  K298:E517  K298:E224  K298:E517  P299:E524  P299:K520  P299:E517  G300:E524  K351:D238  Q420:D185  R460:S236  V461:S236  V467:E192  V467:E192  N468:E192  N468:D189  N468:E192  N468:D189  E470:K196 | R155:K356  E178:R352  T179:R352  T179:L415  T179:S416  R205:T388  R205:E425  R205:E389  R205:E425  K206:T388  A514:R358  E517:R358  E517:R358  **E524:H 420**  **E524:K356**  **E524:H420**  **Q526:S424**  **Q526:P418**  **Q526:V422**  **Q526:S424**  **Y527:S424** |
|  | **Salt bridges** |  |
|  | E115:K579  D117:K572  D117:K576  K272:E425  E280:K521  K298:E224  K298:E517  K351:D238  E470:K196 | R205:E425  R217:E224  E510:R358  E524:K356 |
|  | **Hydrogen bonds** |  |
| 225ns | R160:E535  E166:R33  K298:E224  K298:E517  P299:K520  E332:K521  E470:K196 | K195:D238  R205:E389  R205:E425  **Q526:V422**  **Q526:S424**  **Y527:S424** |
|  | **Non-bonded contacts** |  |
|  | **R156:Y536**  **R160:E535**  **R160:Y536**  **L163:Q36**  **L163:K34**  **E166:R33**  **E166:R33**  **K195:Q36**  **G196:Q36**  K298:E517  K298:E224  K298:E517  K298:E517  P299:K520  P299:E517  E332:K521  V467:E192  E470:K196 | T179:K391  T179:L415  T180:T388  T180:L415  K195:D238  R205:E389  R205:E425  R379:G357  E510:R358  Y511:R358  A514:R358  D518:D419  **Q526:S424**  **Y527:S424** |
|  | **Salt bridges** |  |
|  | R160:E535  E166:R 33  K298:E224  K298:E517  E332:K521  E470:K196 | E178:R352  R205:E389  R205:E425  E510:R358 |
|  | **Hydrogen bonds** |  |
| 250ns | **D117:K576**  **R160:N35**  **R160:Y536**  **R160:K34**  **R160:N35**  **Q253:K572**  E265:K595  Q275:E224  K298:E224  K298:E517  P299:K520  N468:E192  N468:D189  E470:K196 | R205:E389  R205:E425  E517:K356  Q526:V422  Y527:S424 |
|  | **Non-bonded contacts** |  |
|  | D117:K572  D117:K576  **R156:R89**  **R156:Y536**  **E157:E533**  **R160:Y536**  **R160:K34**  **R160:N35**  **R160:N35**  **R160:R53**  Q253:K572  E265:K595  Q275:E224  G276:L228  C277:N225  K298:E517  K298:E224  K298:E517  K298:E224  P299:E524  P299:K520  P299:K521  P299:E517  G300:E524  G300:E524  P333:R573  G465:S236  G465:S236  V467:E192  N468:E192  N468:D189  N468:E192  E470:K196 | E178:R352  T179:R352  T179:L415  T179:S416  T180:T388  R205:E389  R205:E425  R205:E389  R205:E425  E510:R358  A514:R358  E517:K356  Q526:S424  Q526:V422  Q526:P423  Y527:S424 |
|  | **Salt bridges** |  |
|  | D117:K576  K298:E224  K298:E517  E470:K196 | R205:E389  R205:E425  E510:R358  E517:K356  D518:K356 |
| 275ns | **Hydrogen bonds** |  |
|  | D117:K572  R160:Y536  K298:E224  K298:E517  G300:E524  E331:K576  E332:R573  R460:D238  R460:S236  R460:D238  N468:D189  E470:K196 | E178:R352  R205:E389  R205:E425  E517:R358  **E524:K356**  **Q526:V422** |
|  | **Non-bonded contacts** |  |
|  | D117:K572  D117:E575  R156:Y536  R156:R89  R160:R53  R160:Y536  N164:Q36  E166:Q36  K298:E224  K298:E517  K298:E224  K298:E517  P299:E524  P299:K520  P299:E524  G300:E524  E331:K576  E332:R573  R460:S236  R460:D238  P464:S236  G465:S236  V467:E192  V467:K196  N468:E192  N468:D189  R469:F184  R469:D189  E470:K196 | R155:K356  E178:R352  E178:K356  T179:R352  T179:L415  **T179:S416**  R205:T388  R205:E389  R205:E425  K206:T388  Q513:R358  A514:R358  E517:R358  **E524:H420**  **E524:K356**  **Q526:S424**  **Q526:P418**  **Q526:V422**  **Q526:S424**  **Y527:S424** |
|  | **Salt bridges** |  |
|  | D117:K572  K298:E224  K298:E517  E331:K576  E332:R573  R460:D238  E470:K196 | R205:E389  R205:E425  **E524:K356** |
|  | **Hydrogen bonds** |  |
| 300 ns | **R160:K 34**  **R160:E535**  **N164:Q36**  **A165:Q36**  **E166:R33**  **E166:Q36**  K298:E224  K298:E517  G300:E524  E332:R573  R460:D238  R460:D238  R469:T183  E470:K196 | R205:E425  R205:E425  D518:K356  D518:R358  **Q526:V422**  **Q526:S424**  **Y527:S424** |
|  | **Non-bonded contacts** |  |
|  | **R156:Y536**  **R160:K34**  **R160:E535**  **R160:Y536**  **R160:R53**  **R160:K34**  **R160:E535**  **L163:Q36**  **L163:K34**  **N164:Q36**  **A165:Q36**  **E166:Q36**  **E166:R33**  **E166:Q36**  K298:E517  K298:E224  K298:E517  P299:K520  P299:E524  P299:K521  P299:E517  G300:E524  E331:R573  E332:R573  R460:S236  V467:K196  R469:T183  R469:F184  E470:K196 | E178 R352  T179 L415  T180 T388  **R205 E389**  **R205 E425**  **A514 R358**  D518 K356  D518 K356  D518 K356  D518 R358  **E524 G421**  **Q526 S424**  **Q526 V422**  **Y527 S424**  **L533 L228** |
|  | **Salt bridges** |  |
|  | **R160:E535**  **E166: R 33**  K298:E224  K298:E517  E332:R573  R460:D238  E470:K196 | R205:E389  R205:E425  D518:K356  D518:R358 |
