# Supplementary figures and images for: SHP-1 tyrosine phosphatase binding to c-Src kinase phosphor-dependent conformations: A comparative structural framework
Source: PLoS One. 2023 Jan 13;18(1):e0278448. doi: 10.1371/journal.pone.0278448 (PMC9838854; doi:10.1371/journal.pone.0278448)

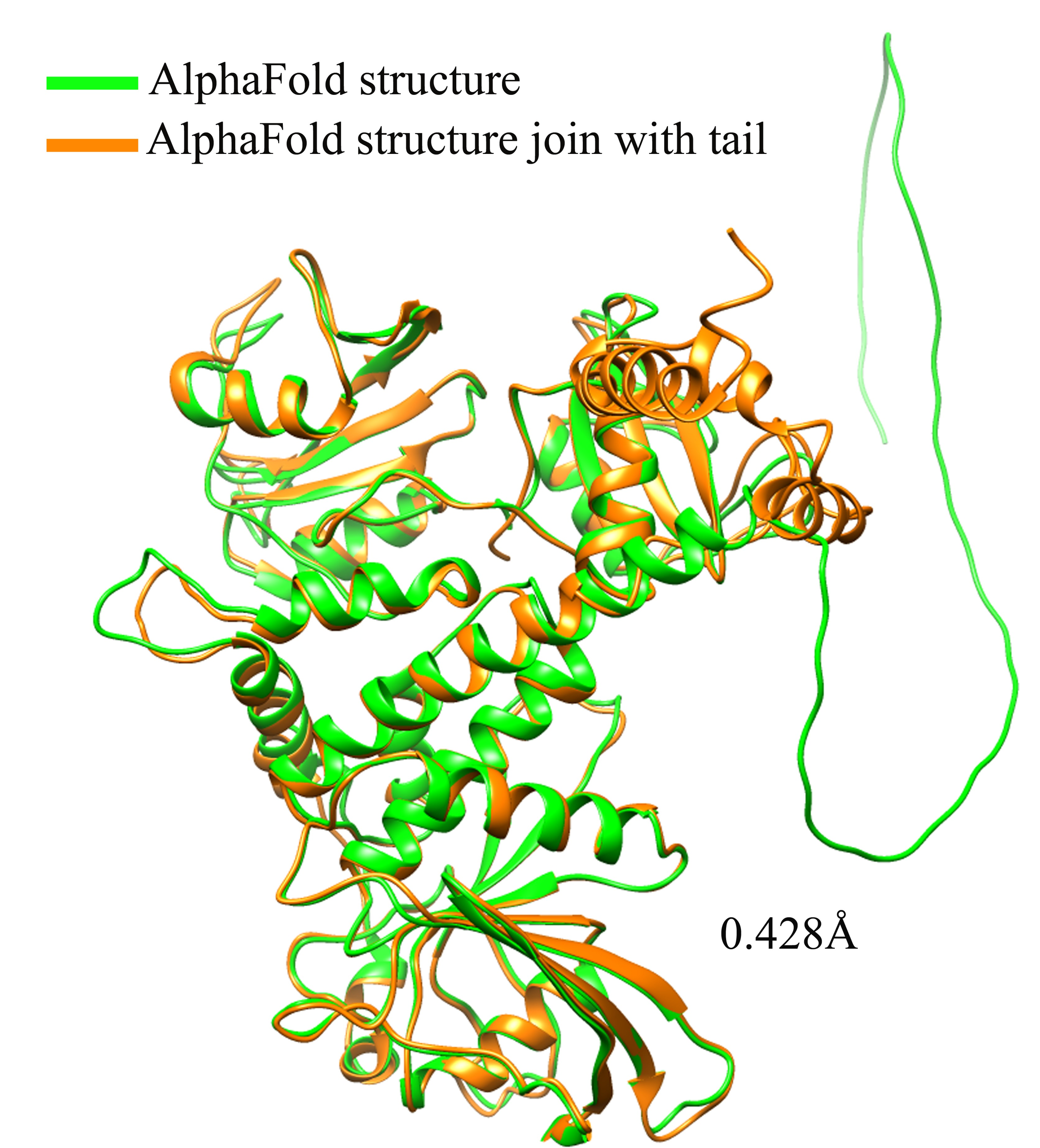

Supplement: S1 Fig — (TIF) [file pone.0278448.s002.tif]

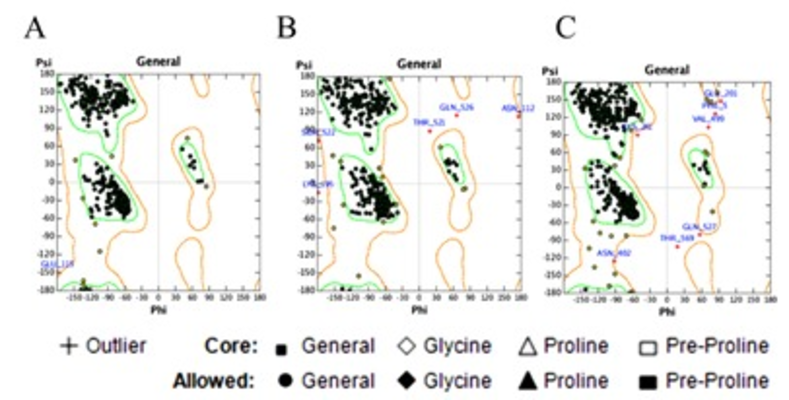

Supplement: S2 Fig — The Ramachandran plot displays the phi-psi torsion angles for (A) c-Srcopen, (B) c-Srcclose and (C) SHP-1. (TIF) [file pone.0278448.s003.tif]

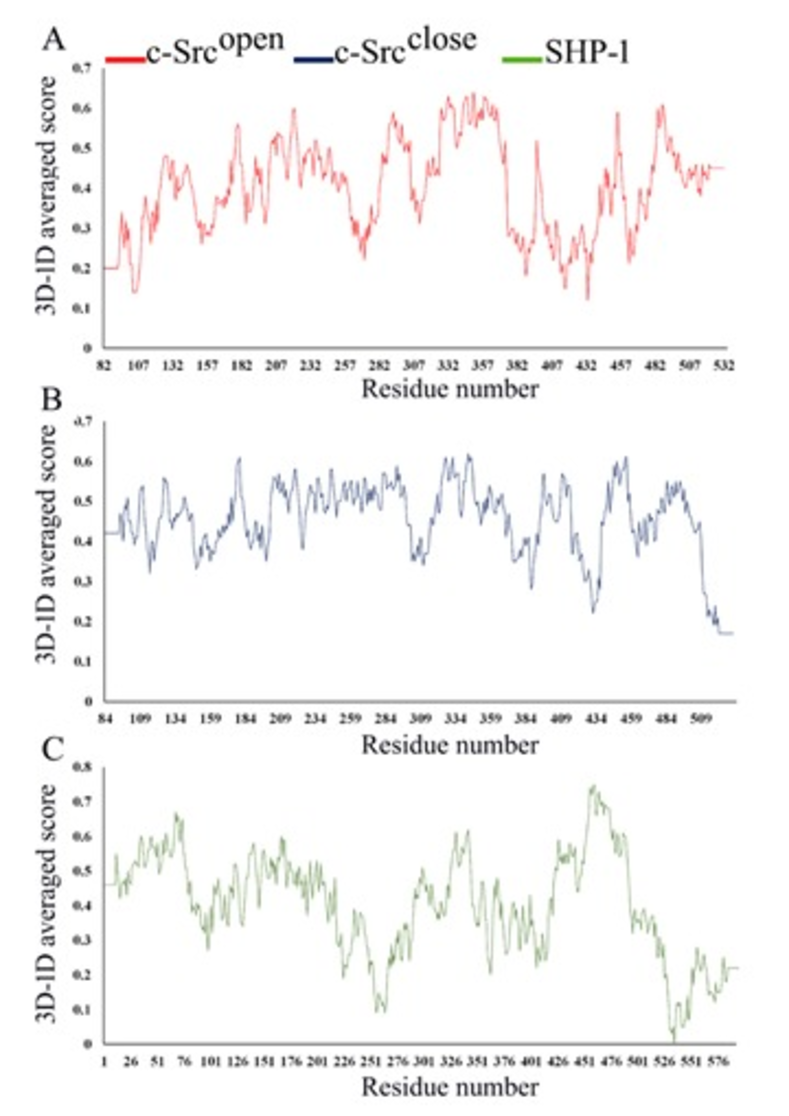

Supplement: S3 Fig — Verify3D plots for (A) c-Srcopen, (B) c-Srcclose, and (C) SHP-1. Y-axis demonstrates the Verify3D score for each residue, whereas the X-axis represents the residue number. (TIF) [file pone.0278448.s004.tif]

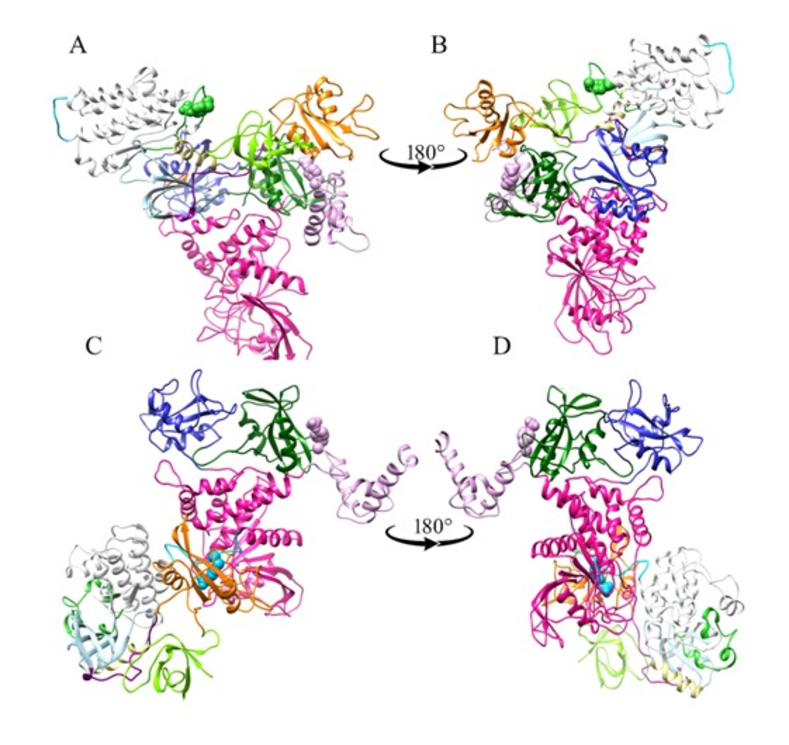

Supplement: S4 Fig — (A and B) c-Srcopen-SHP-1. (C and D) c-Srcclose-SHP-1. SHP-1: N-SH2 in deep green, C-SH2 in deep blue, PTP in pink and C-terminal tail in plum. Open and close conformation of c-Src. Color code: SH3, SH2 domain, and linker region are displayed in chartreuse, orange, and dark magenta colors. Kinase domain, N-lobe, C-lobe, and A-loop are shown in light blue, white, and lime green colors. Gly-loop and C-terminal tail are shown in orange-red and cyan colors, while phosphorylated residues (pY416 and pY527) are shown in the deep pink sphere, respectively. (TIF) [file pone.0278448.s005.tif]

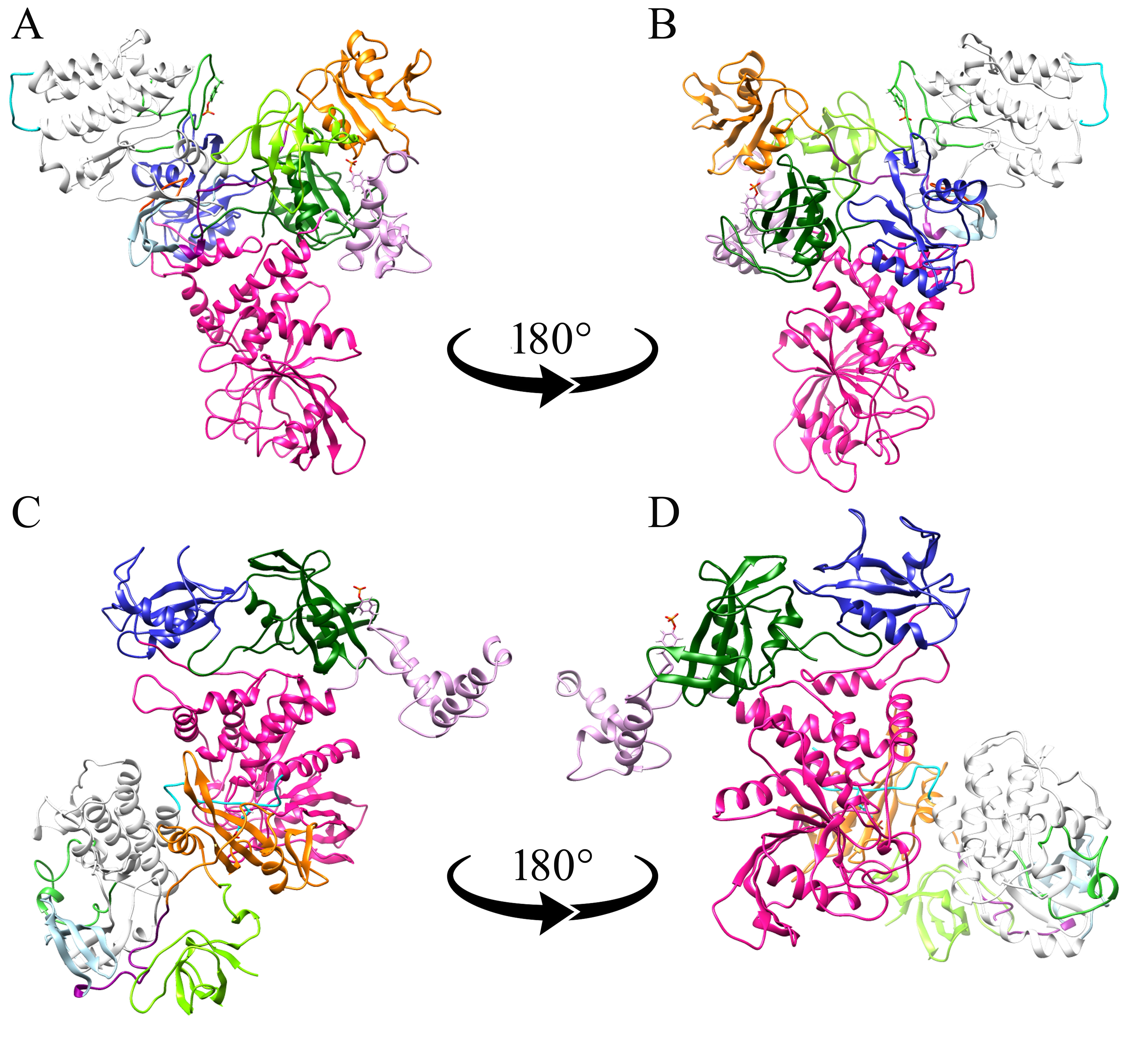

Supplement: S5 Fig — (A and B) c-Srcopen-SHP-1. (C and D) c-Srcclose-SHP-1. SHP-1: N-SH2 in deep green, C-SH2 in deep blue, PTP in pink and C-terminal tail in plum. Open and close conformation of c-Src. Color code: SH3, SH2 domain, and linker region are displayed in chartreuse, orange, and dark magenta colors. Kinase domain, N-lobe, C-lobe, and A-loop are shown in light blue, white, and lime green colors. Gly-loop and C-terminal tail are shown in orange-red and cyan colors, while phosphorylated residues (pY416 and pY527) are shown in the stick, respectively. (TIF) [file pone.0278448.s006.tif]

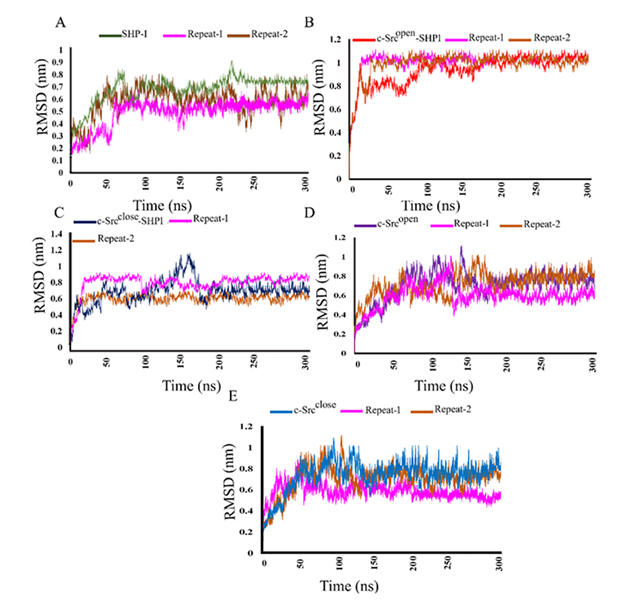

Supplement: S6 Fig — Comparative RMSD for (A) apo-SHP-1 (green), (B) c-Srcopen-SHP-1 (red), (C) c-Srcclose-SHP-1 (blue), (D) c-Srcopen (purple), and (E) c-Srcclose (sky blue), and their replicas are represented in deep pink and orange colors, respectively. (TIF) [file pone.0278448.s007.tif]

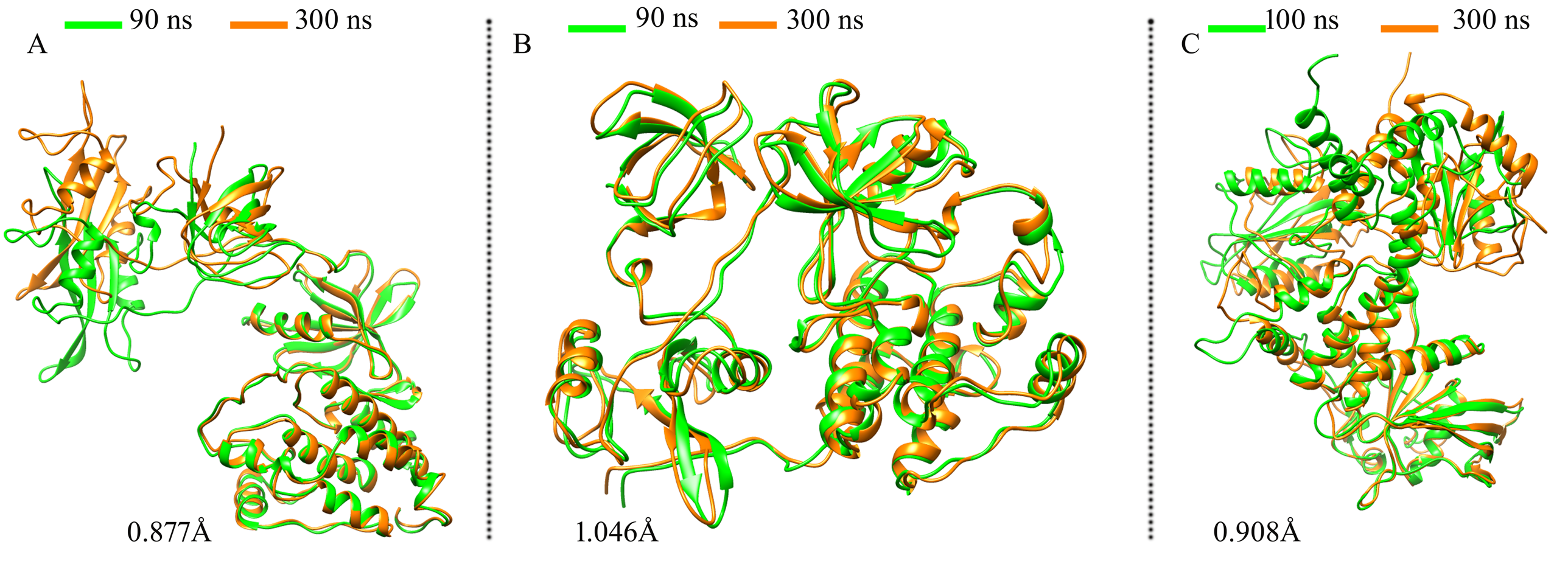

Supplement: S7 Fig — The 90 ns for c-Srcopen, c-Srcclose, and SHP-1 (100ns) are green, while 300 ns are orange. (A) c-Srcopen (B) c-Srcclose (C) SHP1. RMSD values are labeled in angstrom. (TIF) [file pone.0278448.s008.tif]

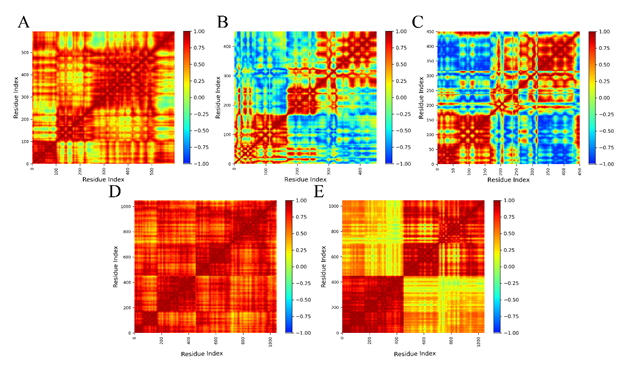

Supplement: S8 Fig — (A) SHP-1, (B) c-Srcopen, (C) c-Srcclose, (D) c-Srcopen- SHP-1, and (E) c-Srcclose-SHP-1 plots. (TIF) [file pone.0278448.s009.tif]
